# The complete mitogenome of *Plator insolens* (Araneae: Trochanteriidae) with phylogenetic implication

**DOI:** 10.1080/23802359.2022.2081098

**Published:** 2022-06-14

**Authors:** Yuhui Ding, Hongru Xu, Huiqin Ma, Junxia Zhang

**Affiliations:** a Key Laboratory of Zoological Systematics and Application of Hebei Province, Institute of Life Science and Green Development, College of Life Sciences, Hebei University, Baoding, Hebei, China; b Hebei Key Laboratory of Wetland Ecology and Conservation, Hengshui University, Hengshui, Hebei, China

**Keywords:** Mitochondrial genome, NGS technique, phylogenetic analysis, RTA clade

## Abstract

*Plator insolens* Simon, 1880 belongs to the family Trochanteriidae and is distributed in China. Herein, we report the complete mitochondrial genome of *P. insolens* reconstructed from Illumina sequencing data, which is the first published mitochondrial genome for the family. The mitogenome is 14,519 bp in length and contains 13 protein-coding genes, 22 transfer RNA genes and two ribosomal RNA genes. The phylogenetic analysis indicates that *P. insolens* is clustered within the RTA clade of the infraorder Araneomorphae. This study provides useful genetic information for future studies on the taxonomy, phylogeny and evolution of trochanteriid species.

*Plator insolens* Simon, 1880, belonging to the family Trochanteriidae, is mainly distributed in Hebei and Henan provinces and occasionally found in Liaoning province of China (Zhu and Zhang 2011). The Trochanteriidae Karsch, 1879 is a relatively small spider family with six genera and 50 species currently known worldwide (World Spider Catalog 2022). Members of this group are commonly known as flattened spiders due to their dorsoventrally flattened bodies which may be an adaptation to life in cracks and under tree barks (Zhu and Zhang 2011; Azevedo et al. 2021). The mitochondrial genomes have provided valuable insights on the evolution of various animal groups (e.g. Nardi et al. 2003; Fujita et al. 2007). However, no complete mitogenome has yet been published for the family Trochanteriidae. Here we performed high-throughput sequencing on a specimen of *P. insolens* from China to determine its mitogenome structure and its phylogenetic relationship with other 19 spider species.

Specimens of *Plator insolens* were collected from Xiong County, Baoding City, Hebei Province, China (38°58′09″N, 116°02′46″E). The spider sampling was permitted by the Institute of Life Science and Green Development, Hebei University (The name and number of the project are the Open Foundation of Hebei Key Laboratory of Wetland Ecology and Conservation, No. hklk201910). The voucher specimen (YHD022) is deposited at the Museum of Hebei University (Yuhui Ding, email: dingyuhui@stumail.hbu.edu.cn). The genomic DNA was extracted with the DNeasy Blood & Tissue Kit (QIAGEN, Hilden, Germany). The sequencing library was produced using the NEXTFLEX Rapid DNA-Seq Kit 2.0 (Bioo Scientific, Austin, USA) and following the manufacturer’s protocol. The prepared library was sequenced on the Illumina Novaseq 6000 platform with 150 bp paired-end reads at Novogene (Tianjin, China). Approximately 4.97 Gbp of raw data were obtained, which were proceeded with quality control to remove reads of low quality (with ≥10% unidentified nucleotides, or with > 50% bases having Phred quality < 5, or with > 10 nt aligned to the adapter, or the read 1 and read 2 of two paired-end reads that were completely identical). The remaining cleaned data were used to assemble the complete mitochondrial genome using Meng et al. (2019). Genome annotation was first performed with the annotation module in Mitoz, and then further polished in the MITOS web server (Bernt et al. 2013). The mitochondrial DNA sequence of *P. insolens* with the annotated genes was deposited in GenBank (accession number: OM397542).

The complete mitogenome of *P. insolens* is circular with 14,519 bp in length. It has 37 mitochondrial genes (13 protein-coding genes, 22 transfer RNAs, and two ribosomal RNA genes) that are typically present in metazoan mitogenomes (Boore 1999). Among the 37 genes, 23 are encoded on the major strand (J-strand) while the others are encoded on the minor strand (N-strand). The gene composition is identical to those found in other spider mitogenomes (Pan et al. 2014; Zhu and Zhang 2017). However, the order of some tRNAs (L: tRNA-Leu, E: tRNA-Glu, A: tRNA-Ala) is apparently different from that of other spider species (Figure 1). The mitogenome of *P. insolens* shows a high nucleotide bias with 71.9% of A + T and 28.1% of G + C (31.3% A; 40.6% T; 19.7% G; and 8.4% C). Among the 13 protein-coding genes (PCGs), most of them start with ATA (COX1, COX3, ND3, ND5, ND4L, ND6, CYTB) or ATT (ND2, ATP6, ND1), and two starts with TTG (ATP8, ND4), and one start with GTG (COX2). Eight PCGs are terminated with TAA (ND2, COX1, ATP6, COX3, ND3, ND4, ND6, ND1). One PCG ends with TAG (COX2) and one ends with an incomplete stop codon (ND6).

**Figure 1.**
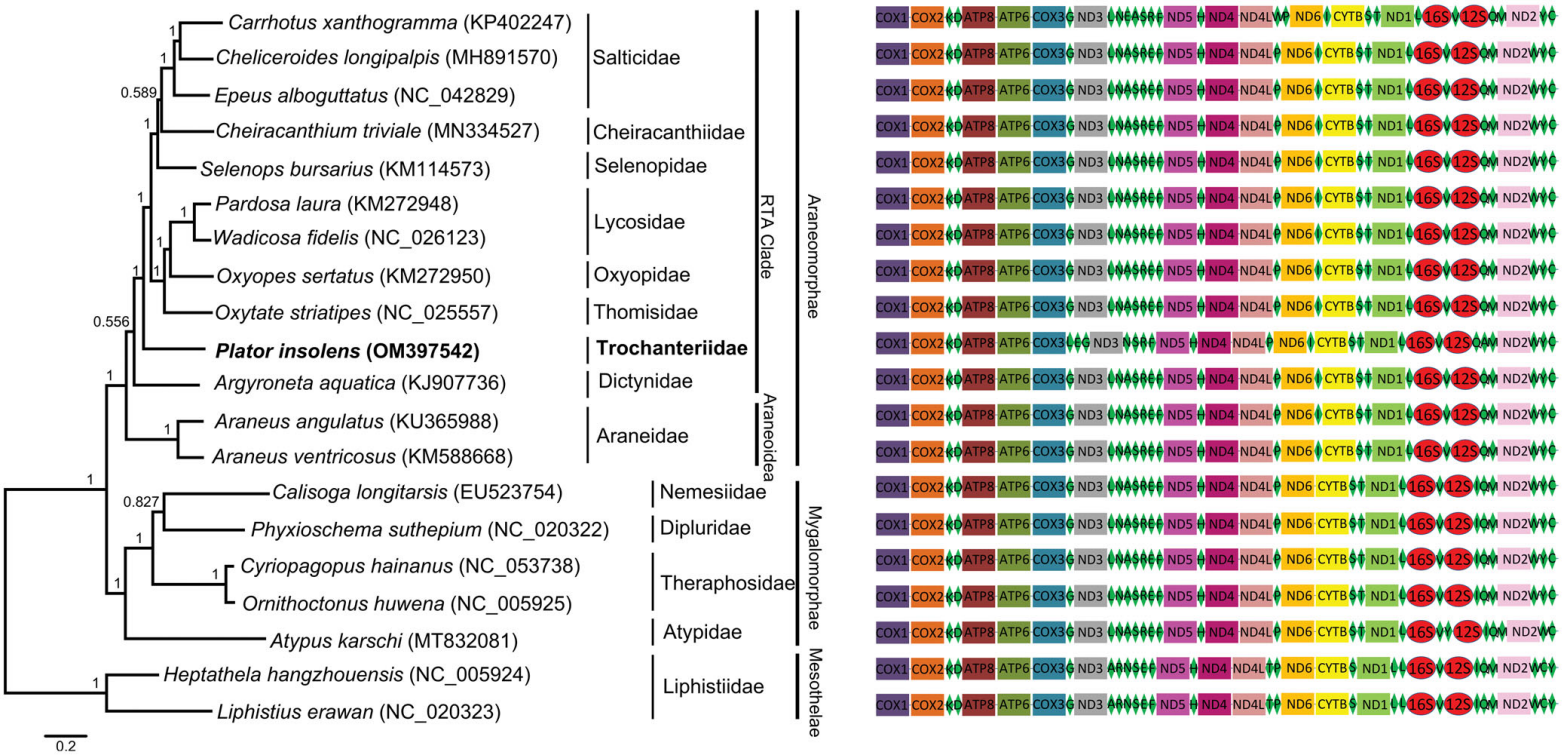
Bayesian phylogenetic analysis of 20 species based on the combined 13 protein-coding genes (numbers along the branch are posterior probabilities). Accession numbers of the mitochondrial sequences used in the phylogenetic analysis are listed in brackets after species. The rectangle, rhombus and ellipse in gene order represent coding genes, tRNA and rRNA respectively.

We constructed the phylogenetic relationships of 20 spider species based on the mitochondrial genome data to test the placement of *P. insolens* in the spider phylogeny. Bayesian inference (BI) based on the nucleotide sequences of the 13 PCGs was conducted using MrBayes 3.2.6 (Ronquist et al. 2012) under the GTR + I + G + F model. The 13 PCGs were extracted and concatenated using PhyloSuite v1.2.1 (Zhang et al. 2020). The phylogenetic tree (Figure 1) shows that *P. insolens* is clustered within the RTA clade of the infraorder Araneomorphae, which is consistent with the result of a recent UCE phylogenomic study (Kulkarni et al. 2021).

## Data Availability

The mitogenome sequence data of *Plator insolens* has been deposited in GenBank of NCBI at https://www.ncbi.nlm.nih.gov/ under the accession no. OM397542. The associated Bio-Project, SRA, and Bio-Sample numbers are PRJNA817674, SRR18355032, and SAMN26804931 respectively.
